# Associations of Insulin Levels and Insulin Resistance With Urine Glucose Excretion Independent of Blood Glucose in Chinese Adults With Prediabetes and Newly Diagnosed Diabetes

**DOI:** 10.3389/fphys.2018.01666

**Published:** 2018-11-21

**Authors:** Juan Chen, Shan-Hu Qiu, Hai-Jian Guo, Wei Li, Zi-Lin Sun

**Affiliations:** ^1^Department of Endocrinology, Zhongda Hospital, Institute of Diabetes, School of Medicine, Southeast University, Nanjing, China; ^2^Integrated Affairs Management Office, Jiangsu Provincial Center for Disease Control and Prevention, Nanjing, China

**Keywords:** diabetes mellitus, glycosuria, urine glucose excretion, insulin, insulin resistance

## Abstract

Several studies have demonstrated that renal glucose reabsorption is increased in patients with type 2 diabetes. However, the increased renal glucose reabsorption may contribute to the progression of hyperglycemia. Therefore, promoting urine glucose excretion (UGE) by suppression of renal glucose reabsorption is an attractive approach for the treatment of diabetes. Insulin resistance is identified as a major characteristic in the pathogenesis of type 2 diabetes. Thus, our aim was to evaluate the association of UGE with serum insulin levels and insulin resistance in subjects with glucose abnormalities, including prediabetes and newly diagnosed diabetes (NDD). The present study included 1129 subjects, 826 individuals with prediabetes and 303 individuals with NDD. Urine samples were collected within 2 h of oral glucose loading for the measurement of glucose. Fasting serum insulin was measured. Homeostatic model assessment of insulin resistance (HOMA-IR) was assessed. Multiple linear regression analysis and multivariate logistic regression analysis were performed to determine the association of UGE with insulin levels and HOMA-IR. A negative association between serum insulin levels and UGE was observed. The relationship remained significant after adjustment for potential confounders, including age, gender, blood pressure and glucose (β = -5.271, 95% CI: -9.775 to -0.767, *p* = 0.022). Furthermore, multivariable logistic regression model showed that increased insulin levels were associated with a decreased risk for high UGE after multivariable adjustment. In addition, similar correlation was also observed between HOMA-IR and UGE. HOMA-IR was negatively correlated with UGE after controlling for potential confounders. Moreover, an independent inverse relationship between HOMA-IR and the risk of high UGE was found (OR = 0.85, 95% CI: 0.78–0.93, *p* < 0.001). In conclusion, insulin levels and HOMA-IR were negatively correlated with UGE after adjusting for potential confounders. Subjects with increased insulin levels or IR were at a decreased risk of high UGE independent of blood glucose. The study suggests that insulin might affect UGE through other ways, in addition to the direct blood glucose-lowering effect, thereby resulting in reduced UGE.

## Introduction

The kidney plays a central role in glucose homeostasis, largely through glucose reabsorption (Gerich, 2010; DeFronzo et al., 2012). Sodium-glucose cotransporters 2 (SGLT2), an important mediator of glucose reabsorption on the luminal surface of proximal tubules, is responsible for more than 90% of glucose reabsorption (Wilding, 2014; Mondick et al., 2016). Accumulating evidences have demonstrated that renal glucose reabsorption is increased in patients with type 2 diabetes mellitus since enhanced SGLT2 expression (DeFronzo et al., 2013; Osaki et al., 2016). However, increased glucose reabsorption may contribute to the progression of hyperglycemia. Therefore, promoting urine glucose excretion (UGE) by inhibition of renal glucose reabsorption has been recognized as an effective strategy for the treatment of diabetes (Ferrannini, 2017). In addition, assessing the significance of UGE in clinical practice, such as glycemic control and diabetes screening, has become a noteworthy field (Lu et al., 2011; Yang et al., 2015; Chen et al., 2018a).

Insulin resistance (IR), a major characteristic in the pathogenesis of type 2 diabetes (Defronzo, 2009), is manifested by increased hepatic glucose production and impaired glucose uptake. Moreover, IR is accompanied by chronic kidney disease (Thomas et al., 2015). Insulin receptor has been found in renal tubular cells, and insulin signaling plays an important role in tubular function (Artunc et al., 2016). Furthermore, it has been reported that insulin can stimulate sodium reabsorption in renal proximal tubules (Baum, 1987; Horita et al., 2016). As is well known, the transport of glucose from the tubular lumen into tubular cells is sodium dependent. Accordingly, insulin may play an important role in glucose reabsorption in the renal proximal tubular cells. To date, few studies have focused on the association between insulin levels and renal glucose reabsorption. Renal threshold for glucose reabsorption can reflect the capacity of renal glucose reabsorption. However, gold-standard stepwise hyperglycemic clamp procedure (SHCP) method cannot be widely used in the clinical practice to estimate renal threshold for glucose reabsorption, because of specialized laboratory demand. However, it is easy to obtain data on UGE.

Therefore, the aim of the present study was to investigate the association of serum insulin levels and IR with UGE in subjects with glucose abnormalities, including prediabetes and diabetes.

## Materials and Methods

### Study Design and Participants

Data were obtained from a cross-sectional study undertaken to evaluate the efficacy of UGE in diabetes screening in Chinese population, aged 18–65 years and without previously diagnosed diabetes or taking anti-diabetic medication (Chen et al., 2018b). This study was approved by Ethics Review Committee of Jiangsu Provincial Center for Disease Control and Prevention and followed the tenets the Declaration of Helsinki. Written informed consent was obtained from each participant. All participants were given a standard 75 g glucose solution. All the urine samples were collected over a 2 h period after oral glucose loading for quantitative measurement of urine glucose. Finally, after confirming their oral glucose tolerance test and excluding those who had no data on fasting serum insulin level, 1129 subjects with glucose abnormalities including prediabetes and newly diagnosed diabetes (NDD) were included in the present study.

### Anthropometric and Laboratory Measurements

Demographic characteristics and medical histories were obtained using a structured questionnaire. Weight, height, heart rate (HR), and blood pressure (BP) were measured according to standardized protocols. Body mass index (BMI) was calculated as weight in kilograms divided by height in meters squared. Fasting plasma glucose (FPG) and 2 h plasma glucose (2h-PG) were measured with the glucose oxidase method using an automatic chemistry analyzer (Synchron LX-20, Beckman Coulter Inc., CA, United States). The concentration of urinary glucose was measured with a quantitative urine meter (UG-201-H, Tanita Corporation, Tokyo, Japan). UGE was calculated as the urinary glucose concentration (mg/dl) × the urine volume (dl). Fasting serum insulin level was evaluated by electrochemiluminescence immunoassay (Roche Diagnostics, Indianapolis, IN, United States). Insulin resistance was estimated by homeostasis model assessment for insulin resistance (HOMA-IR): FPG (mmol/L) × fasting insulin (mIU/L)/22.5.

### Definitions

Prediabetes and newly diagnosed diabetes were defined according to the 1999 World Health Organization (WHO) criteria. In our previous study, UGE displayed an excellent sensitivity of 82.9% and a high specificity of 84.7% in detecting NDD at the corresponding optimal cutoff of 130 mg (Chen et al., 2018a). Therefore, in the present study, UGE exceeding 130 mg was considered as high UGE, while UGE less than 130 mg was considered as low UGE.

### Statistical Analysis

The continuous variables in this study were presented as the means ± SD or median (25th–75th percentiles) as appropriate. The categorical variables were presented as numbers (%). The differences between the groups were analyzed using independent Student’s *t*-tests for normally distributed variables, non-parametric Mann–Whitney *U*-tests for non-normally distributed variables and chi-square test for categorical data. The relationships between UGE and other clinical indicators was examined using Spearman’s correlation. Multiple linear regression analysis with adjustment for potential confounders was conducted to access the association of insulin levels and IR with UGE. Potential confounders included in the multivariate analysis were age, gender, HR, BP, FPG, 2h-PG, total cholesterol (TC), triglycerides (TG), high-density lipoprotein-cholesterol (HDL-c), low-density lipoprotein-cholesterol (LDL-c), creatinine, blood urea nitrogen (BUN), and BMI, which were chosen depend on reaching statistical significance in the Spearman’s correlation analysis and based on clinical judgment. A binary logistic regression analysis was performed to determine the odds ratios of high nUGE associated with insulin levels and IR. A *P*-value < 0.05 was considered statistically significant. All statistical analyses were performed using SPSS 22.0 (SPSS Inc., Chicago, IL, United States).

## Results

### General Characteristics of the Study Participants

A total of 1129 subjects, including 826 individuals with prediabetes and 303 individuals with NDD, were included in the present study. The general characteristics of the study population, according to UGE, were summarized in Table 1. Subjects with high UGE exhibited significant higher BP, FPG, 2h-PG, TC, TG, and BMI compared with those with low UGE. In addition, no significant differences in age, HR, insulin, HOMA-IR, HDL-c, LDL-c, creatinine, and BUN were found between the two groups.

**Table 1:** Baseline characteristics of the study participants according to UGE.

	Low UGE^a^	High UGE^b^	*P*
Number	586 (51.9%)	543 (48.1%)	
Age (years)	48.60 ± 11.08	48.62 ± 9.75	0.977
Male (%)	243 (41.5%)	318 (58.6%)	<0.001
HR (beats/min)	78.62 ± 12.15	79.47 ± 12.12	0.241
**BLOOD PRESSURE (mmHg)**			
Systolic	135.15 ± 19.83	138.66 ± 19.30	0.003
Diastolic	82.46 ± 11.67	85.33 ± 11.51	<0.001
**PLASMA GLUCOSE (mmol/L)**			
FPG	5.94 ± 0.70	7.00 ± 1.86	<0.001
2h-PG	7.95 ± 1.78	10.90 ± 4.19	<0.001
Insulin (mIU /L)	6.90 (4.80–10.30)	6.60 (4.20–9.50)	0.053
HOMA-IR	1.79 (1.25–2.66)	1.98 (1.23–3.01)	0.066
TC (mmol/ L)	4.91 ± 0.99	5.06 ± 0.97	0.010
TG (mmol/ L)	1.41 (0.96–2.05)	1.67 (1.11–2.36)	<0.001
HDL-c (mmol /L)	1.38 ± 0.39	1.35 ± 0.36	0.102
LDL-c (mmol /L)	2.79 ± 0.78	2.88 ± 0.77	0.054
Creatinine (umol/L)	71.45 ± 15.76	72.68 ± 16.73	0.205
BUN (mmol/L)	5.21 ± 1.51	5.36 ± 1.45	0.086
BMI (kg/m^2^)	26.10 ± 3.79	26.80 ± 3.74	0.002

*Data are presented as n (%), mean ± SD, or median (25th–75th percentiles) as appropriate.^a^UGE < 130 mg. ^b^UGE ≥ 130 mg. HR, heart rate; FPG, fasting plasma glucose; 2h-PG, 2 h plasma glucose; TC, total cholesterol; TG, triglycerides; HDL-c, high-density lipoprotein–cholesterol; LDL-c, low-density lipoprotein–cholesterol; BUN, blood urea nitrogen; LUGE, low urine glucose excretion; HUGE, high urine glucose excretion; BMI, body mass index.*

### Correlations of UGE With Other Clinical Indicators

Spearman’s correlation showed that UGE was positively related to FPG and 2h-PG, whereas negatively correlated with serum insulin levels (*r* = -0.063, *p* = 0.034). Moreover, significant positive correlations of UGE with HR, BP, HOMA-IR, TG, TC, LDL-c, BUN, and BMI were observed (Table 2).

**Table 2:** The correlations of UGE with other clinical indicators in subjects with glucose abnormalities.

	UGE	
	*R*	*P-*value
Age	0.010	0.736
HR	0.068	0.022^∗^
Systolic BP	0.127	<0.001^∗^
Diastolic BP	0.148	<0.001^∗^
FPG	0.468	<0.001^∗^
2h-PG	0.468	<0.001^∗^
Insulin	-0.063	0.034^∗^
HOMA-IR	0.071	0.017^∗^
TC	0.077	0.009^∗^
TG	0.146	<0.001^∗^
HDL-c	-0.083	0.005^∗^
LDL-c	0.063	0.035^∗^
BUN	0.076	0.011^∗^
Creatinine	0.030	0.308
BMI	0.110	<0.001^∗^

*Data are presented as r (P-value).*

*^∗^Significance, p < 0.05. UGE, urine glucose excretion; HR, heart rate; BP, blood pressure; FPG, fasting plasma glucose; 2h-PG, 2-h plasma glucose; HbA1c, glycated hemoglobin; TC, total cholesterol; TG, triglycerides; HDL-c, high-density lipoprotein cholesterol; LDL-c, low-density lipoprotein cholesterol; BUN, blood urea nitrogen; BMI, body mass index.*

### Multiple Linear Regression Analysis With UGE as the Dependent Variable

To identify the association of UGE with serum insulin levels and HOMA-IR, and eliminate the influence of confounders, multiple linear regression analysis with UGE as a dependent variable was presented in Table 3. HOMA-IR and insulin were analyzed in separate models due to collinearity. Insulin levels were negatively associated with UGE after adjustment for potential confounders, including age, gender, FPG, 2h-PG, systolic and diastolic blood pressure, and BMI (β = -5.271, 95% CI: -9.775 to -0.767, *p* = 0.022). The males were more likely to have higher UGE than females after multivariable adjustment. In addition, FPG and 2h-PG were still positively associated with UGE in this model. Moreover, a negative association between HOMA-IR and UGE was observed after controlling for other variables (β = –4.767, 95% CI: -28.477 to -1.057, *p* = 0.035).

**Table 3:** Multiple linear regression analyses with UGE as the dependent variable.

Independent variable	b Coefficient	95% CI	Standardized coefficient	*P*
**MODEL 1**				
Age (years)	-1.663	-4.858 to 1.532	-0.026	0.307
Gender^a^	-292.210	-363.542 to -218.877	-0.217	<0.001
FPG (mmol/L)	95.187	68.392 to 121.982	0.210	<0.001
2h-PG (mmol/L)	87.996	76.713 to 99.279	0.459	<0.001
Insulin (mIU/L)	-5.271	-9.775 to -0.767	-0.056	0.022
Systolic (mmHg)	0.939	-1.271 to 3.150	0.027	0.405
Diastolic (mmHg)	2.576	-1.066 to 6.219	0.045	0.165
TC (mmol/L)	2.746	-45.363 to 50.855	0.004	0.911
TG (mmol/L)	10.585	-5.982 to 27.152	0.034	0.210
BMI (kg/m^2^)	5.367	-3.261 to 13.996	0.030	0.223
**MODEL 2**				
Age (years)	-1.521	-4.703 to 1.662	-0.024	0.349
Gender^a^	-291.520	-363.876 to -219.164	-0.217	<0.001
FPG (mmol/L)	99.721	72.044 to 127.397	0.220	<0.001
2h-PG (mmol/L)	88.229	76.952 to 99.505	0.460	<0.001
HOMA-IR	-14.767	-28.477 to -1.057	-0.052	0.035
Systolic (mmHg)	0.892	-1.318 to 3.102	0.026	0.428
Diastolic (mmHg)	2.671	-0.969 to 6.312	0.046	0.150
TC (mmol/L)	2.724	-45.403 to 50.851	0.004	0.912
TG (mmol/L)	10.421	-6.152 to 26.994	0.034	0.218
BMI (kg/m^2^)	4.881	-3.687 to 13.449	0.028	0.264

*Both models were adjusted for age, gender, heart rate, blood pressure, FPG, 2h-PG, TG, TC, high-density lipoprotein–cholesterol, low-density lipoprotein–cholesterol, creatinine, blood urea nitrogen, and body mass index. ^*a*^1 = male, 2 = female. UGE, urine glucose excretion; FPG, fasting plasma glucose; 2h-PG, 2 h plasma glucose; TC, total cholesterol; TG, triglycerides; BMI, body mass index; HOMA-IR, homeostasis model assessment for insulin resistance.*

### Logistic Regression Analysis of Odds Ratio for High UGE

Furthermore, a binary logistic regression analysis was performed to identify the factors associated with odds ratios of high UGE. Increasing FPG and 2h-PG were significantly associated with an increased odds ratio of HUGE in the multi-adjusted model (Table 4). However, increased serum insulin levels were significantly associated with a decreased odds ratio of high UGE (OR = 0.96, 95% CI: 0.93–0.98, *p* < 0.001). In addition, the data also showed an independent inverse relationship between the HOMA-IR and the risk of high UGE (OR = 0.85, 95% CI: 0.78–0.93, *p* < 0.001).

**Table 4:** Multiple logistic regression analyses of odds ratios for high UGE.

Category	β	SE of β	OR	95% CI	*P*-value
**MODEL 1**					
FPG (mmol/L)	0.920	0.112	2.51	2.02–3.12	<0.001
2h-PG (mmol/L)	0.393	0.037	1.48	1.38–1.60	<0.001
Insulin (mIU/L)	-0.046	0.013	0.96	0.93–0.98	<0.001
**MODEL 2**					
FPG (mmol/L)	0.989	0.117	2.69	2.14–3.38	<0.001
2h-PG (mmol/L)	0.396	0.037	1.49	1.38–1.60	<0.001
HOMA-IR	-0.160	0.042	0.85	0.78–0.93	<0.001

*Both models were adjusted for age, gender, heart rate, blood pressure, FPG, 2h-PG, TG, TC, high-density lipoprotein–cholesterol, low-density lipoprotein–cholesterol, creatinine, blood urea nitrogen, and body mass index.*

*SE, standard error; OR, odds ratio; CI, confidence interval; FPG, fasting plasma glucose; 2h-PG, 2 h plasma glucose; UGE, urine glucose excretion; HOMA-IR, homeostasis model assessment for insulin resistance.*

## Discussion

Studies on the associations of serum insulin levels and HOMA-IR with UGE are relatively scarce. Previous studies have demonstrated that insulin stimulates sodium reabsorption and the transport of glucose is sodium dependent in renal proximal tubules (Baum, 1987; Ferrannini, 2017). Thus, we hypothesized that insulin may participate in renal glucose reabsorption, which may have influence on UGE. In the present study, we found that serum insulin levels were negatively associated with UGE. The relationship remained significant after adjustment for age, gender, heart rate, blood pressure, FPG, 2h-PG, TG, TC, HDL-c, LDL-c, creatinine, BUN, and BMI (Table 3). Furthermore, multivariable logistic regression model showed that insulin levels were associated with a decreased risk of high UGE. Similar correlation was also observed between HOMA-IR and UGE. The study established that increased insulin levels and HOMA-IR were strongly correlated with decreased risk of high UGE after controlling for other variables, indicating that insulin might reduce UGE independent of blood glucose.

Glycosuria is the result of glycemic excursions in excess of the renal glucose threshold (Osaki et al., 2016). Much more attention has been paid to the significance of UGE on health and disease, such as glycemic control and diabetes screening (Lu et al., 2011; Dallosso et al., 2015; Yang et al., 2015). However, factors associated with UGE have not been elucidated clearly in patients with diabetes. UGE increases in a proportional manner with increasing blood glucose (Rave et al., 2006). Consistent with previous studies (Rave et al., 2006; Yang et al., 2015), positive relationships of UGE with FPG and 2h-PG were observed in the present study. In addition, our data showed that both increased insulin levels and HOMA-IR were significantly associated with a decrease in the risk of high UGE. The study by Ono et al. (2017) also revealed an inverse association between the insulinogenic index and UGE. However, this study was conducted in subjects with prediabetes. Our study population was consisted of participants with prediabetes and NDD. Furthermore, a recent study reported that individuals with increased HOMA-IR were at an increased risk for high renal threshold for glucose reabsorption (Yue et al., 2017), suggesting that subjects with IR are more likely to have enhanced renal glucose reabsorption, which may cause a reduction in UGE. Taken together, we found insulin levels and IR were negatively associated with UGE independent of blood glucose, and further suggested that in addition to glucose-lowering action, as a major polypeptide hormone, insulin may affect UGE in other ways.

SGLT2, a highly specific and major glucose transporter in kidney tubules, is responsible for more than 90% of tubular glucose reabsorption (Nair and Wilding, 2010; Ferrannini, 2017). Overexpression of SGLT2 has been observed in both animal models and humans with diabetes (Rahmoune et al., 2005; Vallon et al., 2013). Obviously, increased glucose reabsorption may contribute to the progression of hyperglycemia (Wilding, 2014). Several studies have demonstrated the efficacy of SGLT2 inhibitors for the improvement of glucose control by inhibiting glucose reabsorption and increasing UGE (Devineni et al., 2012). As is well known, insulin resistance is one of the major characteristics in the pathogenesis of type 2 diabetes. Besides, insulin receptor has been found in renal tubular cells. A recent study found the deletion of insulin receptor significantly reduced SGLT2 expression and increased UGE, the study suggested that insulin, rather than glucose, may primarily regulate SGLT2 abundance and glucose transport (Nizar et al., 2018). In addition, another study demonstrated that insulin could stimulate SGLT-2-mediated glucose entry into proximal tubular cells (Nakamura et al., 2015). Taken together, elevated insulin levels may be a major factor to influence the expression of SGLT2 and UGE. Therefore, subjects with elevated insulin levels or HOMA-IR were more likely to have low UGE independent of blood glucose, may be attributed to enhanced glucose reabsorption via upregulation of SGLT2.

To date, few studies have focused on the association of insulin levels and IR with UGE. Our study population is consisted of subjects with no history of previous diabetes or taking any antidiabetic medication. Therefore, the correlation between insulin and UGE may be more accurate due to the elimination of the impacts of antidiabetic medication on UGE or insulin levels. However, some limitations should be noticed in this study. First, individuals with increased insulin levels and IR were at a decreased risk of high UGE independent of blood glucose, which might be attributed to increased renal glucose reabsorption. However, renal glucose reabsorption was not evaluated in this study. Future studies are needed to evaluate the renal threshold of glucose reabsorption in subjects with hyperinsulinemia or IR. In addition, further study is necessary to confirm whether hyperinsulinemia may induce SGLT2 overexpression in humans or animal models. Second, most subjects with normal glucose tolerance did not have obvious UGE. There is no significant correlation between insulin levels and UGE in subjects with normal glucose tolerance. Our study only involved subjects with prediabetes and NDD, so that the present findings may not be extrapolated to subjects with normal glucose tolerance or previous history of diabetes. Third, the causal relationships could not be deduced in the cross-sectional study. Finally, we did not measure insulin levels 30, 60, and 90 min post-glucose loading. Nevertheless, the present study could still provide valuable information to help us understand the association of UGE with insulin.

## Conclusion

In conclusion, increased serum insulin levels and HOMA-IR were associated with a decreased risk of high UGE independent of blood glucose in subjects with glucose abnormalities, which might be attributed to the increased renal glucose reabsorption.

## Data Availability

The raw data supporting the conclusions of this manuscript will be made available by the authors, without undue reservation, to any qualified researcher.

## Author Contributions

Z-LS and JC were responsible for the study design. JC was responsible for data collection, data analysis, interpretation, and writing of the manuscript. S-HQ, H-JG, and WL were responsible for data collection and assisted with data interpretation and writing. All authors read the manuscript critically and approved the submitted version.

## Conflict of Interest Statement

The authors declare that the research was conducted in the absence of any commercial or financial relationships that could be construed as a potential conflict of interest.
